# High-Efficiency Classification of White Blood Cells Based on Object Detection

**DOI:** 10.1155/2021/1615192

**Published:** 2021-09-13

**Authors:** Jiangfan Yao, Xiwei Huang, Maoyu Wei, Wentao Han, Xuefeng Xu, Renjie Wang, Jin Chen, Lingling Sun

**Affiliations:** Key Laboratory of RF Circuits and Systems, Ministry of Education, Hangzhou Dianzi University, Hangzhou 310018, China

## Abstract

White blood cells (WBCs) play a significant role in the human immune system, and the content of various subtypes of WBCs is usually maintained within a certain range in the human body, while deviant levels are important warning signs for diseases. Hence, the detection and classification of WBCs is an essential diagnostic technique. However, traditional WBC classification technologies based on image processing usually need to segment the collected target cell images from the background. This preprocessing operation not only increases the workload but also heavily affects the classification quality and efficiency. Therefore, we proposed one high-efficiency object detection technology that combines the segmentation and recognition of targets into one step to realize the detection and classification of WBCs in an image at the same time. Two state-of-the-art object detection models, Faster RCNN and Yolov4, were employed and comparatively studied to classify neutrophils, eosinophils, monocytes, and lymphocytes on a balanced and enhanced Blood Cell Count Dataset (BCCD). Our experimental results showed that the Faster RCNN and Yolov4 based deep transfer learning models achieved classification accuracy rates of 96.25% and 95.75%, respectively. For the one-stage model, Yolov4, while ensuring more than 95% accuracy, its detection speed could reach 60 FPS, which showed better performance compared with the two-stage model, Faster RCNN. The high-efficiency object detection network that does not require cell presegmentation can remove the difficulty of image preprocessing and greatly improve the efficiency of the entire classification task, which provides a potential solution for future real-time point-of-care diagnostic systems.

## 1. Introduction

White blood cells (WBCs) are important parts of human blood and indispensable security guards in the immune system, which mainly include granulocytes, monocytes, and lymphocytes [1]. Granulocytes are differentiated from hematopoietic stem cells in the bone marrow. They are a type of leukocytes containing granules in the cytoplasm and can be divided into three subtypes: neutrophils, eosinophils, and basophils, according to their morphological characteristics under Wright's staining (see Table 1). Neutrophils are composed of multisplit nuclei containing between two and five lobes. They are the most common phagocytes, accounting for 50%–60% of the total number of WBCs. The content of eosinophils is between 1% and 6%, and there are generally two split nuclei. Basophils are one of the least common cells in bone marrow and blood, and their content is less than 2%. Monocytes are produced in the bone marrow and are a kind of WBCs with nongranular cytoplasm, accounting for 2%–10% of whole WBCs. Lymphocytes are the smallest type of WBCs, which can be divided into T cells and B cells. They have almost no cytoplasm, accounting for about 20%–30%. These WBC populations have characteristic concentration ranges in healthy people. Many diseases are accompanied by their concentration deviations [2], such as inflammation and bacterial infection. Therefore, the classification and statistics of WBCs have important medical diagnostic significance.

Traditional WBC classification is achieved by experienced medical personnel, who directly differentiated the WBCs from blood smear images according to their morphologies under the microscope [3]. Manual classification has many shortcomings and difficulties. For example, human observation cannot guarantee unbiased estimation, which may lead to unsatisfactory accuracy. Moreover, manual classification is time-consuming, complicated, and requires strict professional skills of the inspectors, which cannot meet the requirements of high-efficiency classification tasks on large scales nowadays. Therefore, automatic WBC classification technologies have been extensively developed [4, 5].

Existing automatic classification technologies are mostly based on cell image analysis because they are relatively easy to obtain, and different WBC types show distinctive morphological characteristics. In addition, image processing technologies have matured in recent years with the help of advancing computing power and intelligent algorithms. The two most representative technologies are feature engineering based on machine learning [6, 7] and automatic feature extraction based on deep learning [8, 9].

**Figure 1:**
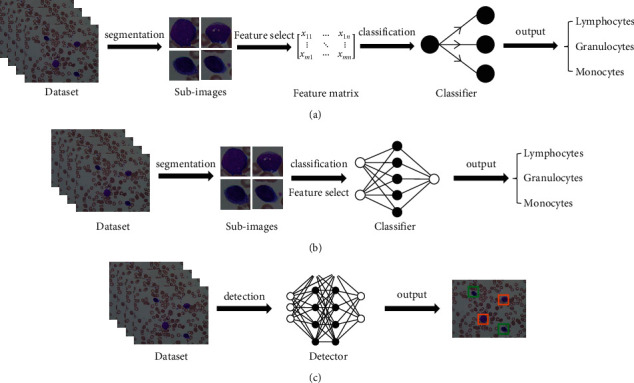
Three WBC classification systems: (a) feature engineering classification system based on machine learning; (b) automatic feature extraction classification system based on deep learning; and (c) high-efficiency object detection classification system based on deep learning.

The feature engineering classification system based on machine learning mainly follows three steps: (I) target segmentation from the background, (II) manual extraction of effective or unique features, and (III) classifier design (see Figure 1(a)). This method uses a shallow machine learning model that relies on input features and attempts to quantify the relevant features extracted from digital images with an analysis method similar to morphologists; then, it uses them as the input of the prediction algorithm. Shallow machine learning methods commonly used in classification generally include support vector machine (SVM), naive Bayes classifier, linear discriminant analysis (LDA), and multilayer perceptron (MLP).

For example, Lippeveld et al. studied the WBC classification performance of classical machine learning with manually selected feature values as input and obtained 0.776 mean average precision (mAP) on unstained WBCs captured by the imaging flow cytometer (IFC) [10]. Nassar et al. used a gradient boosting algorithm to achieve the label-free classification of four types of WBCs (neutrophils, eosinophils, monocytes, and lymphocytes) and two types of lymphocytes (B/T lymphocytes) based on IFC, and the *F*1 scores reached 97% and 78%, respectively [11]. However, the focus of these studies is mostly on image preprocessing (target segmentation) and feature selection, which are the prerequisites for the good performance of the classification system.

Deep learning is a technology widely used in research fields such as computer vision, speech analysis, and natural language processing (NLP) [12]. As a data-based representation learning algorithm, deep learning uses supervised or semisupervised feature learning and hierarchical extraction algorithms to replace manual feature acquisition. The automatic feature extraction system based on deep learning can be divided into two steps: (I) image preprocessing (cell segmentation, data enhancement) and (II) neural network design (see Figure 1(b)).

For example, in our previous work, we used a fine-tuned ResNet50 network to realize the label-free classification of WBCs and neutrophils of different activation states, reaching an accuracy of more than 90% [13]. Chen et al. proposed a high-throughput quantitative imaging system using photon time stretching and achieved high-precision classification of label-free cells (T lymphocyte and colon cancer cells) using deep learning algorithms [14]. Shu et al. used quantitative phase microscopy to image unstained leukocytes and used the CNN model to extract cell morphological features to achieve the classification of four types of WBCs [15] with 90% accuracy obtained in both training set and test set.

The above two classification systems are designed from the perspective of image classification. They must first segment the objects from the backgrounds to ensure that there are as few irrelevant backgrounds as possible in the image input to the network. The idea of object segmentation is to obtain the pixel block containing the target from the original image by setting a threshold, and the intercepted area only contains at most one target. When the image background is not much different from the cells, or the image has excessive noise, the difficulty of cell segmentation will be greatly increased. Moreover, the performance of the classification system depends heavily on the preprocessing of cell segmentation, which makes it difficult to achieve end-to-end optimization of the entire system. By contrast, object detection is an algorithm based on target geometric and statistical features, which combines the tasks of target segmentation and recognition into one process. While finding the precise location of a specific object in a given image, a corresponding class label is also assigned to each object instance (see Figure 1(c)). This algorithm combining classification and localization does not need to perform object segmentation in advance; hence, it effectively solves the problems of the above two systems.

Current object detection algorithms can be divided into two-stage models and one-stage models. The two-stage models first use a latent region generator to generate a sparse proposal region and extract features from each proposal and then cascade a region classifier to predict the candidate region labels. The most representative two-stage detectors are regional convolution neural networks (RCNN) [16], Fast RCNN [17], Faster RCNN [18], and Mask RCNN [19]. By contrast, the one-stage models directly classify and predict the objects at each location of the feature map, so that cascaded region classification is not required. The representative models of one-stage detectors include YOLO [20] and SSD [21]. In theory, a two-stage model can achieve a high accuracy rate but has more parameters and a slower detection speed. A one-stage model can achieve a speed increase but usually at the expense of accuracy.

For example, Kutlu et al. [22] tested several RCNN models with different backbone networks on randomly mixed Blood Cell Count Dataset (BCCD) and Leukocyte Images for Segmentation and Classification (LISC) dataset. The best performance is achieved on ResNet50, and the highest accuracy is obtained in the detection of lymphocytes (99.52%). However, the detection speed of this series RCNN is low, only 1.6 FPS. Wang et al. [23] collected high-resolution blood cell images with a Fourier engraving microscope and successfully detected WBCs using the Yolov3 model. The accuracy and recall rate reached 100%, achieving better performance than other algorithms. However, their work is based on high-resolution images collected by a unique imaging system, and they can only identify WBCs from the background but cannot complete the classification task. Wang et al. [24] used the Yolov3 fine-tuning model to achieve the detection of multiple types of WBCs at a speed of 53 FPS, but this model could be further optimized. Jiang et al. [25] proposed an Attention-YOLO for the detection and counting of WBC, red blood cells, and platelets, but its dataset is imbalanced and the processing efficiency in terms of frame rate is not analyzed and compared.

To achieve leukocyte classification with optimized speed and accuracy, we employed the most state-of-the-art one-stage model Yolov4 to achieve detection of neutrophils, eosinophils, monocytes, and lymphocytes on an enhanced and balanced BCCD for training. We adopted the transfer learning mechanism and adjusted the training parameters such as batch size and learning rate so that the models could achieve classification accuracy rates of 95.75% and detection speed of 60 FPS. As a comparison with Yolov4, we also employed the Faster RCNN on the same dataset. By applying different backbone networks (ResNet101, VGG16, Inception v2), we found that VGG16 converged faster (similar to Yolov4, about 100 epoch), and finally obtained 96.25% accuracy and 15 FPS detection speed. It can be seen that Yolov4 is more efficient, with comparable accuracy in terms of classifying such WBC images. Therefore, the proposed high-efficiency one-stage detection network provides a potential solution for future real-time point-of-care (POC) diagnostic systems [26, 27].

## 2. Materials and Methods

### 2.1. Data Enhancement and Annotation

The original dataset used in our experiment is BCCD (https://www.kaggle.com/paultimothymooney/blood-cells), which contains 364 microscope images stained by the Reiter-Giemsa staining method. It is a small-scale dataset for blood cell detection, which includes WBCs, RBCs, and platelets in each image. The nuclear characteristics of the stained WBCs are relatively obvious, and four subtypes of WBCs (neutrophils, eosinophils, monocytes, and lymphocytes) can be identified as our classification task. From the perspective of cell morphology, T and B lymphocytes are indistinguishable [28]. Both of their cells have a large nucleus with dense heterochromatin. Therefore, we do not distinguish these two subtypes of lymphocytes. In addition, the proportion of basophils in the human body is very small (<2%), which has little reference value in pathology, so we also ignore it.

To a large extent, the performance of a deep learning algorithm requires a large enough dataset in the training phase to avoid overfitting. However, in the field of medical image analysis, it is difficult to obtain adequate raw data for CNN training [29]. Data augmentation is an image processing technology that solves the limited data space, which can improve the size and quality of training data so that it can be used to build better deep learning models. Image enhancement algorithms include geometric transformation, color space enhancement, mixed images, and neural style transfer [30]. In this paper, we mainly use rotation and mirror geometric transformation algorithms to expand the BCCD and improve the performance of our model:

(1) Central rotation: in reality, the origin of the image is in the upper left corner, so we need to move the origin of the upper left corner to the center of the image. Suppose a point on the image (*X*_0_, *Y*_0_), the image width is *W*, the height is *H*, the image rotation angle is *θ* (to avoid the loss of WBC information in the image, here we choose a smaller value of *θ*), the transformed point is (*X*_1_, *Y*_1_), the width and height of the target image are *W*^*∗*^ and *H*^*∗*^, respectively; then, we can get the following transformation formula:
  (1) $$\begin{array}{c} \left[\begin{array}{c} X_{1}\\ Y_{1}\\ 1 \end{array}\right]=\left[\begin{array}{ccc} 1 & 0 & 0\\ 0 & -1 & 0\\ -0.5W & 0.5H & 1 \end{array}\right]\left[\begin{array}{ccc} \cos\;\theta & -\sin\;\theta & 0\\ \sin\;\theta & \cos\;\theta & 0\\ 0 & 0 & 1 \end{array}\right]\left[\begin{array}{ccc} 1 & 0 & 0\\ 0 & -1 & 0\\ -0.5W^{*} & 0.5H^{*} & 1 \end{array}\right]\left[\begin{array}{c} X_{0}\\ Y_{0}\\ 1 \end{array}\right]. \end{array}$$
(2) Mirror flip: we used two mirroring methods here, horizontal mirroring and vertical mirroring. Horizontal mirroring takes the vertical centerline of the image as the axis, swapping the pixels of the image, while vertical mirroring takes the horizontal centerline of the image as the axis and reverses the upper half and the bottom half of the image. Therefore, the transformation formula for horizontal mirroring is
  (2) $$\begin{array}{c} X_{1}=W-X_{0}-1,\\ Y_{1}=Y_{0}. \end{array}$$

The vertical mirror transformation formula is

(3) $$\begin{array}{c} Y_{1}=H-Y_{0}-1,\\ X_{1}=X_{0}. \end{array}$$

**Figure 2:**
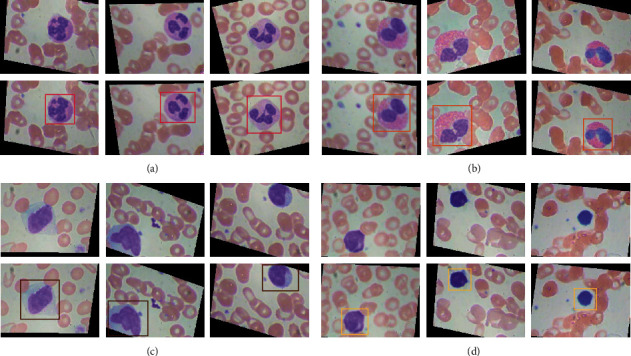
Samples (top) and their corresponding annotations (bottom) of the four WBC subtypes in our dataset. (a) Neutrophil, (b) eosinophil, (c) monocyte, and (d) lymphocyte.

We randomly selected 225 images of four types of WBCs from the enhanced images to construct our dataset, and the image resolution is 320 × 240. Then, according to the 7 : 2 ratio, it is divided into a training set (175 images of each of the four types of WBCs) and a test set (50 images of each of the four types of WBCs). The image after the rotation and mirroring process has black borders, but this will not affect our classification task, because the object detection only pays attention to the information of the marked target (localization box and class label), which will not be affected by the background. We used *labelImg* (https://github.com-/tzutalin/labelImg) to generate the corresponding annotation files for the training and testing later. Examples of image samples and annotations are shown in Figure 2.

## 3. Detectors

### 3.1. Faster RCNN

**Figure 3:**
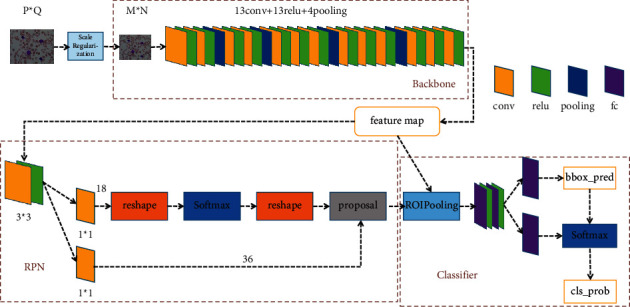
Faster RCNN architecture. The backbone is VGG16 without a fully connected layer used to extract the features of entire images to generate the feature map, RPN is used to generate the detection box of region proposal, and classifier network is used to classify the candidate detection box and output the detection result.

Faster RCNN [18] is the most outstanding product of the RCNN series algorithms so far, and it is also the most classic object detection algorithm in the two-stage models. It abandons the traditional Sliding Window and Selective Search algorithms, which directly uses the Region Proposal Network (RPN) to generate the detection box, achieving end-to-end training and greatly speeding up. The first stage of Faster RCNN is to find the anchor boxes of the object to be detected in the images (classification of the background and the object), and the second stage is to classify and regress these anchor boxes. The employed Faster RCNN structure based on the VGG16 [31] backbone is shown in Figure 3, which is composed of three parts: backbone, regional proposal network (RPN), and classifier.

The output of RPN and classification regression network is coordinate regression and classification values, so the Loss function of these two networks can be expressed as follows:

(4) $$\begin{array}{c} L\left(\left\{p_{i}\right\},\left\{t_{i}\right\}\right)=\frac{1}{N_{\text{cls}}}\underset{i}{\sum }L_{\text{cls}}\left(p_{i},p_{i}^{*}\right)+\frac{\lambda }{N_{\text{reg}}}\underset{i}{\sum }p_{i}^{*}L_{\text{reg}}\left(t_{i},t_{i}^{*}\right), \end{array}$$

where *i* represents the anchor index, *p*_*i*_ represents the probability of positive class that Softmax outputs, and *p*_*i*_^*∗*^ represents the corresponding ground truth prediction probability. When the IoU between the *i*th  anchor and ground truth is greater than 0.7, this anchor is considered to be positive (*p*_*i*_=1). Otherwise, when IoU < 0.3, the anchor is considered to be negative (*p*_*i*_=0). *t*_*i*_^*∗*^ represents the prediction probability of the bounding box. *t*_*i*_^*∗*^ represents the prediction probability of the ground true corresponding to the positive anchor. *L*_cls_ represents the classification loss function (used the anchor's classification in training), which can be used for different loss functions. *L*_reg_ represents the regression loss function (used the bounding box regression in training), which uses smooth_*L*1_ here. The calculation is as follows:

(5) $$\begin{array}{c} L_{\text{reg}}\left(t_{i},t_{i}^{*}\right)=\sum_{i\varepsilon \left(x,y,w,h\right)}{\text{smooth}}_{L1}\left(t_{i}-t_{i}^{*}\right),\\ {\text{smooth}}_{L1}\left(x\right)=\left\{\begin{array}{cc} 0.5x^{2}, & \text{if }\left|x\right|<1,\\ \left|x\right|-0.5, & \text{otherwise.} \end{array}\right.\; \end{array}$$

In the actual process, the gap between *N*_cls_ and *N*_reg_ is too large, so the parameter *λ* is introduced to balance them (e.g., when *N*_cls_=512, *N*_reg_=2400, set *λ*=*N*_reg_/*N*_cls_ ≈ 5). Therefore, the total Loss can be balanced between the two kinds of Loss.

### 3.2. Yolov4

**Figure 4:**
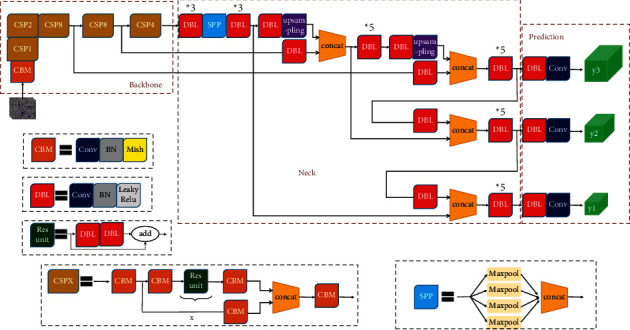
Yolov4 architecture. The backbone is CSPDarknet53 used to feature extraction, the Neck layer (usually between backbone and output) is used to collect feature maps in different stages to better extract fusion features, and the prediction module is used for the output detection result.

Yolo (you only look once) was proposed by Redmon et al. [20] in 2016. As the pioneering work of the one-stage detector, it combines the region proposal generation and candidate box regression into one network and directly predicts the bounding box and class probabilities from the complete image to achieve end-to-end optimization of the entire detection performance. Redmon continued to optimize the algorithm in the next few years and finally developed it into three versions: Yolo, Yolov2 [32], Yolov3 [33]. Yolov3 is an end-to-end one-stage detector with excellent performance, which is comparable to the most advanced object detection system in terms of accuracy and greatly improves the detection speed. Inspired by Yolov3, Bochkovskiy et al. [34] proposed an improved version of Yolov3 in 2020 and named it Yolov4. A large number of experiments have proved that compared with Yolov3, Yolov4 improves the mAP by 10 points on the MS COCO dataset and increases the speed by 12%. In the previous preexperiment, we used Yolov3 and Yolov4 to train the same dataset and found that the Yolov4 fits faster, but the loss is only 1/10 of Yolov3's loss. The network structure of Yolov4 follows the classic one-stage structure, which is mainly divided into three parts: Backbone, Neck, and Prediction. The overall architecture is shown in Figure 4.

**Figure 5:**
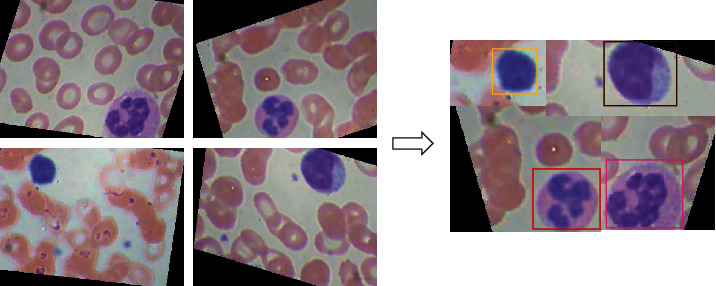
Mosaic data enhancement.

Building on the CutMix [35] algorithm, Yolov4 introduced Mosaic data enhancement at the input. The main idea is to randomly flip, zoom, gamut the color, and crop the four pictures and then stitch them in a picture as training data (see Figure 5). The cutting process will keep the complete label frame. Four pictures are calculated at the same time during batch normalization so that the minibatch size does not need to be large, reducing computing resources. In our dataset, a training sample contains only two WBCs to be detected at most, so the mosaic algorithm can greatly enrich the background of the images and make the network more robust.

Like Faster RCNN, the loss function of Yolov4 is generally composed of two parts: a classification loss function and a regression loss function. Starting from Faster RCNN applying smooth_*L*1_ Loss to bounding box regression, the development of the loss function has gone through IoU Loss (2016), GIoU Loss (2019), DIoU Loss (2020), and now CIoU Loss (2020) applied in Yolov4. IoU Loss mainly considers the overlapping area of the detection box and ground truth. GIoU Loss solves the problem when the bounding boxes do not coincide based on IoU. DIoU Loss considers the information of the center point distance of the bounding box based on IoU and GIoU. CIoU Loss adds an impact factor *αv* to the DIoU's penalty item, which takes into account the aspect ratio of the predicted box to fit the ground truth:

(6) $$\begin{array}{c} R_{\text{CIoU}}=\frac{\rho^{2}\left(b,b^{\text{gt}}\right)}{c^{2}}+\alpha v, \end{array}$$

where *b* and *b*^gt^ represent the center points of the penalty terms of the prediction box *B* and the ground truth *B*^gt^, respectively; *ρ*(·) represents the Euclidean distance; *c* represents the diagonal distance of the smallest outer rectangle of *B* and *B*^gt^; and *α* is the parameter for trade-off, which is defined as

(7) $$\begin{array}{c} \alpha =\frac{v}{\left(1-\text{IoU}\right)+v}, \end{array}$$

where IoU represents the intersection ratio between the prediction box and the ground truth and *v* is a parameter used to measure the consistency of the aspect ratio, which is defined as

(8) $$\begin{array}{c} v=\frac{4}{\pi^{2}}\left(\arctan\frac{w^{gt}}{h^{gt}}-\arctan\frac{w^{2}}{h}\right). \end{array}$$

Therefore, the CIoU Loss function is defined as

(9) $$\begin{array}{c} L_{\text{CIoU}}=1-\text{IoU}+R_{\text{CIoU}}\\ =1-\text{IoU}+\frac{\rho^{2}\left(b,b^{\text{gt}}\right)}{c^{2}}+\alpha v. \end{array}$$

In this way, CIoU Loss can consider three important geometric factors for the box regression function, overlap area, center point distance, and aspect ratio, which improves the speed and accuracy of prediction box regression.

## 4. Results and Discussion

The methods based on Faster RCNN and Yolov4 are implemented using TensorFlow and PyTorch frameworks, respectively, which configure the CUDA8.0 toolkit with cuDNN6.0 library on the 64-bit Ubuntu 16.04 operating system. All experiments are performed with the configuration of CPUi7-6700 (3.40 GHz), RAM16 GB, GPU NVIDIA GTX1080Ti (11 GB), and Python 2.7. In the training phase, we adopted a transfer learning mechanism: initialize the CNN framework with a pretrained model based on the ImageNet (http:/www.image-net.org) and then use our dataset to refine all layers of the network.

We used precision and recall to evaluate the performance of our object detection model, which are defined as follows:

(10) $$\begin{array}{c} \text{precision}=\frac{\text{TP}}{\text{TP}+\text{FP}}\\ =\frac{\text{TP}}{\text{all detections}},\\ \text{recall}=\frac{\text{TP}}{\text{TP}+\text{FN}}\\ =\frac{\text{TP}}{\text{all groundtruth}}. \end{array}$$

where TP (true positive) means correct detection of the positive bounding box; FP (false positive) means false detection of nonexistent objects or misplacement detection of existing objects; and FN (false negative) means missing detection of the positive bounding box. The precision evaluates the ability of models to identify related objects, and the recall rate evaluates the ability of models to find all relevant positive bounding boxes.

**Figure 6:**
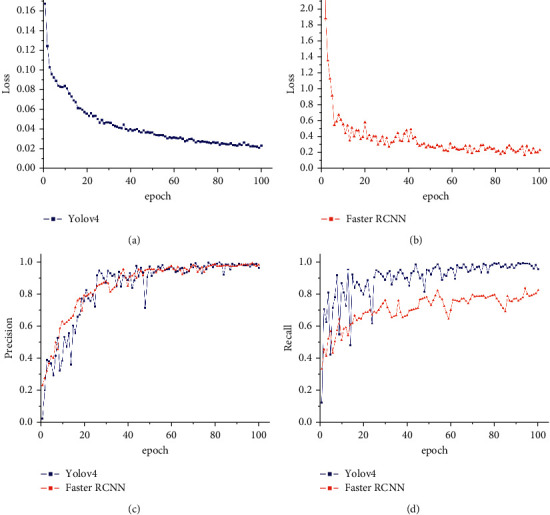
Training output of Yolov4 and Faster RCNN. After 100 epochs, Faster RCNN's loss value curve (a) drops to 0.2, while Yolov4's loss value (b) drops to 0.02. Yolov4's precision and recall rate curve fluctuate widely, and Faster RCNN's precision curve is smoother, but the recall rate is about 10 percentage points lower than Yolov4's (c, d).

We trained the two models for 100 epochs and evaluated their training loss, precision, and recall rate, as shown in Figure 6. We found that the training loss of both models will decay as the number of training iterations increases. The training loss of the Yolov4 model fluctuates less and the convergence is small (Figure 6(a)), while the training precision of Faster RCNN fluctuates slightly but its recall rate is more than 10% lower than that of Yolov4 (Figures 6(c) and 6(d)). It took 0.65 hours of training time for the Yolov4 to fit the model, which was 0.215 times that of Faster RCNN. It can be seen that Yolov4 is lighter and requires less computing power, so easier to integrate into miniaturized equipment.

**Figure 7:**
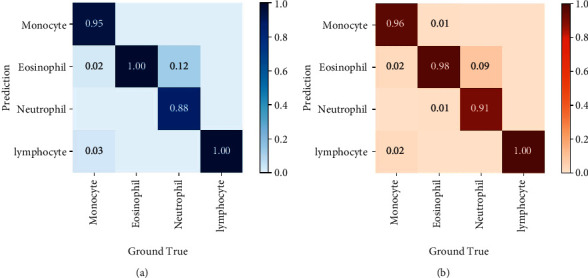
Confusion matrix of (a) Yolov4 and (b) Faster RCNN.

We verified both models on the test set and obtained the confusion matrix of the four types of WBCs (see Figure 7). In the confusion matrix, we can see that both models have achieved great performance. The classification accuracy rates reached 95.75% and 96.25%, respectively. In general, the classification effects of lymphocytes and eosinophils were the best, and the classification effect of neutrophils was the worst. Both models tend to recognize some neutrophils as eosinophils. Both neutrophils and eosinophils are granulocytes, and the difference in nucleus morphology was not significant compared with other types of WBCs. The identification of such subtle differences has always been the driving force for the continuous development of object detection. The detection of the specific dataset (such as raw staining-free WBCs image by self-build imaging system) may require special modifications to the network, such as increasing the depth of feature extraction backbone and optimizing the recognition of small targets, which is our main direction in the future research.

**Figure 8:**
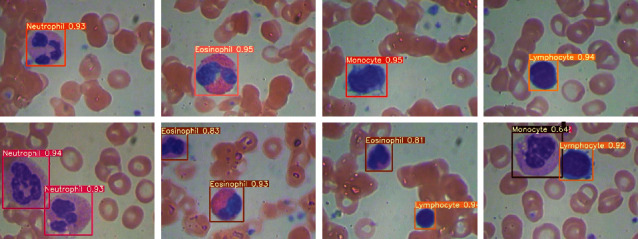
The inference performance of the Yolov4 model on unseen images.

Finally, we used the weights obtained from Yolov4 training to infer the original untrained image in the BCCD. As a result, the model took about 16 ms to detect an image, which is about 60 FPS. It was proved that Yolov4 had a great classification and recognition effect for the four WBC subtypes (see Figure 8). It could also achieve excellent detection for some cells that were closely spaced or located at the edges. The confidence of all inference results is mostly above 0.8.

## 5. Conclusions and Future Perspectives

WBC classification is essential in medical diagnostics as hematologists can estimate a patient's immune condition using accurate WBC classification technology. The traditional manual classification method is limited by many factors, and the automatic WBC classification technology based on computer vision has gradually developed. Feature engineering classification systems based on machine learning and automatic feature extraction classification system based on deep learning are the two most representative technologies. However, both of them need to presegment abundant cells from the background, and only one single cell can be classified at a time; this greatly reduces processing efficiency. As a result, the complexity of the entire system is increased and the effect of classification strictly depends on image preprocessing.

In this paper, we described the superiority of using high-efficiency object detection for WBC classification compared to classic classification methods with preprocessing of single-cell segmentation. We comparatively applied two object detection models to classify the four subtypes of WBCs on an enhanced BCCD with more than 95% classification accuracy. Through the comparative experiments, we have verified that Yolov4 achieves excellent detection speed (up to 60 FPS) while ensuring high accuracy compared to Faster RCNN (about 15 FPS), which provides a favorable reference for real-time WBC classification at the POC. Therefore, it is potentially feasible to use one-stage object detection models in a microfluidic imaging system, that is, no preprocessing work; for example, cell segmentation is required to directly classify WBCs on an image containing multiple cells.

In future research, we intend to apply the Yolov4 to classify the staining-free WBCs that have blurrier morphological features and no obvious nuclei. Based on this study, our envisioned plan to optimize the Yolov4 based model is to increase the depth of the backbone network to extract smaller granularity features and strengthen the feature fusion algorithm of the Neck network so that the detection performance of small WBC targets can be improved. Meanwhile, we will also consider the overall efficiency of the network, obtaining the trade-off between accuracy and speed.

## Figures and Tables

**Table 1:** 3D models, normal concentration ranges, and morphological characteristics for various subtypes of WBCs.

Subtype of WBC		3D model [1]	Content (%)	Morphological characteristics
Granulocytes	Neutrophil	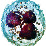	50∼60	Consist of a multilobed nucleus; the number of lobes can be 2–5; stained in natural pink color.
	Eosinophil	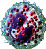		Have a two-lobed nucleus; stained in brick-red in acidic stains
	Basophil	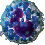		Contain large cytoplasmic granules which obscure the cell nucleus under the microscope when stained.

Monocytes		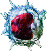	2∼10	Amoeboid in appearance; have nongranulated cytoplasm.

Lymphocytes	B-cell	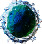	20∼30	Have a large, dark-staining nucleus with little cytoplasm; a coarse and dense nucleus approximately the size of a red blood cell (RBC).
	T-cell	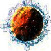		

## Data Availability

Some or all data, models, or codes generated or used during the study are available on GitHub (https://github.com/Ricoshallow) following funder data retention policies.
